# Development of the Japanese Version of the Alzheimer's Disease Cooperative Study—Activities of Daily Living in Mild Cognitive Impairment Scale

**DOI:** 10.1111/psyg.70232

**Published:** 2026-10-20

**Authors:** Daiki Ishimaru, Maki Suzuki, Hideki Kanemoto, Kunihiko Katsuki, Yuma Nagata, Natsuho Hirakawa, Daiki Taomoto, Yuto Satake, Rei Takahashi, Kazue Shigenobu, Kenji Yoshiyama, Manabu Ikeda

**Affiliations:** ^1^ Department of Medical Technology The University of Osaka Hospital Suita Osaka Japan; ^2^ Department of Psychiatry The University of Osaka Graduate School of Medicine Suita Osaka Japan; ^3^ Department of Behavioural Neurology and Neuropsychiatry The University of Osaka United Graduate School of Child Development Suita Osaka Japan; ^4^ Health and Counselling Centre The University of Osaka Toyonaka Osaka Japan; ^5^ Department of Psychiatry Higashikouri Hospital Hirakata Osaka Japan; ^6^ Department of Psychiatry Nippon Life Hospital Osaka Osaka Japan; ^7^ Department of Psychiatry Asakayama General Hospital Sakai Osaka Japan; ^8^ Brain and Mind Future Medical Center Osaka Psychiatric Medical Center Hirakata Osaka Japan

**Keywords:** activities of daily living, Alzheimer's disease, mild cognitive impairment, psychometric properties

## Abstract

**Background:**

Assessment scales to detect subtle declines in activities of daily living (ADL) among patients with mild cognitive impairment (MCI) are needed for early diagnosis and support. This study examined the psychometric properties of the Japanese version of the Alzheimer's Disease Cooperative Study‐Activities of Daily Living for Mild Cognitive Impairment (ADCS‐MCI‐ADL‐J), an ADL assessment scale adapted for patients with early‐stage Alzheimer's disease (AD).

**Methods:**

This multisite study recruited patients with MCI due to AD or mild AD dementia (ADD), and healthy controls (HC) between March 2024 and March 2026. Reliability (internal consistency, inter‐rater reliability, and test–retest reliability) and validity (discriminative, concurrent, convergent, and construct validities) were analysed as psychometric properties. As an interpretability analysis, we also checked the score distributions of the ADCS‐MCI‐ADL‐J to investigate a ceiling effect compared with other existing ADL assessment scales.

**Results:**

A total of 102 participants were included in the analysis (HC, *n* = 30; MCI due to AD, *n* = 37; mild ADD, *n* = 35). Sixty participants (58.8%) were female and the overall mean age (standard deviation) was 72.6 (9.1). The Cronbach's *α* for the ADCS‐MCI‐ADL‐J was 0.893, indicating acceptable internal consistency. The inter‐rater and test–retest reliabilities were excellent, indicated by intraclass correlation coefficients of 0.917 and 0.915, respectively. Areas under the curve were 0.937 for MCI versus HC and 0.859 for MCI versus mild ADD. The ADCS‐MCI‐ADL‐J significantly distinguished MCI due to AD from HC and mild ADD. The ADCS‐MCI‐ADL‐J score was significantly correlated with other ADL scales and the severity of dementia, cognitive impairment, and neuropsychiatric symptoms. The ADCS‐MCI‐ADL‐J was divided into three factors. The ADCS‐MCI‐ADL‐J scores showed a wider distribution without a ceiling effect compared to other existing scales.

**Conclusions:**

This study examined several psychometric properties of ADCS‐MCI‐ADL‐J. This assessment scale is useful for reliably detecting subtle declines in ADL at the MCI stage.

**Trial Registration:**

UMIN‐CTR: UMIN000055015

## 1 Introduction

Although patients with mild cognitive impairment (MCI) are generally considered to maintain independence in overall activities of daily living (ADL), a growing body of evidence suggests that patients with MCI due to Alzheimer's disease (AD) exhibit a subtle decline in instrumental ADL (IADL) [1, 2]. These declines often involve complex tasks, such as financial management and food preparation, while basic ADL are largely preserved. As the disease progresses to AD dementia (ADD), difficulties with basic ADL (BADL), i.e., dressing or bathing gradually emerge [1]. ADL assessment scales that can detect subtle declines in ADL at milder stages are needed to support early diagnosis and timely intervention.

Several ADL assessment scales have been developed for patients with MCI, including the Alzheimer's Disease Cooperative Study—Activities of Daily Living for Mild Cognitive Impairment (ADCS‐MCI‐ADL) scale [3], Amsterdam IADL Questionnaire [4], and Instrumental Activities of Daily Living—Compensation scale [5]. These have demonstrated good ability to distinguish the difficulties in ADL between patients with MCI and those with dementia in the absence of a ceiling effect [5, 6, 7], suggesting that they can capture subtle declines associated with MCI. Many previous studies, including clinical trials, have used these scales for clinical evaluations or as outcome measures [8].

Despite the availability of these MCI‐specific scales, the Physical Self‐Maintenance Scale (PSMS) and Lawton IADL scale are widely used in Japan to assess ADL in patients with MCI [9]. The Disability Assessment for Dementia (DAD) scale has also been used to assess functional impairment in this population [10]. However, these scales were not originally developed for patients with MCI and include few items sensitive to functional decline at the MCI stage, potentially limiting their ability to capture specific impairments observed in patients with MCI.

Among MCI‐specific scales, the ADCS‐MCI‐ADL has been widely used clinically and in clinical trials to assess ADL in patients with MCI worldwide [3]. We previously translated the ADCS‐MCI‐ADL into Japanese (ADCS‐MCI‐ADL‐J) and reported its clinical usefulness in preliminary analysis [11]. In that study, PSMS and Lawton IADL scores were mostly distributed in the higher range among patients with MCI due to AD, whereas ADCS‐MCI‐ADL scores were more widely distributed [11]. Hence, the ADCS‐MCI‐ADL‐J is suggested to be useful for ADL assessment in MCI without a ceiling effect. However, the psychometric properties of the ADCS‐MCI‐ADL‐J have not been fully examined. Therefore, this study aimed to evaluate the reliability and validity of this scale in patients with AD‐induced MCI due to AD.

## 2 Methods

### 2.1 Study Design

This cross‐sectional observational study was conducted at the Department of Psychiatry of the University of Osaka Hospital, Asakayama Hospital, Nippon Life Hospital, and Higashikouri Hospital between March 11, 2024 and March 24, 2026. This study was conducted in accordance with the principles of the 1975 Declaration of Helsinki, revised in 2013, and approved by the University of Osaka Clinical Research Review Committee [23 274(T4)‐8]. The study protocol was registered with UMIN‐CTR (UMIN000055015).

### 2.2 Participants

The inclusion criteria were: (1) diagnosis of MCI due to AD or ADD according to the National Institute on Aging‐Alzheimer's Association 2011 criteria [12, 13], (2) Clinical Dementia Rating (CDR) global score of 0.5 or 1, and (3) age ≧ 40 years. The exclusion criterion was any severe or unstable physical/psychiatric comorbidity that could significantly impair cognition or physical performance.

Caregivers, such as spouses or family members of patients undergoing examination or treatment in the study setting, were also recruited as healthy control (HC) participants, provided they met the following criteria: (1) age ≧ 40 years, (2) minimal subjective memory complaints, indicated by Everyday Memory Checklist (EMC) score ≦ 14 for age < 60 years, or score ≦ 16 for ≥ 60 years [14], (3) met the predefined cognitive criteria based on Mini‐Mental State Examination‐Japanese (MMSE‐J) (score > 27) [15] and Wechsler Memory Scale‐Revised Logical Memory II scores [score ≧age‐standard value of 1.5 standard deviation (SD)] [16, 17], and (4) preserved IADL function, as defined as a gender‐specific full Lawton IADL score (5 points for men and 8 points for women).

The exclusion criteria were severe and unstable physical or psychiatric comorbidities that could impair cognition or physical performance.

### 2.3 Sample Size

According to the Consensus‐based Standards for the Selection of Health Status Measurement Instruments (COSMIN) checklist, a sample of 30–49 participants was considered fair for reliability testing [18]. We planned to recruit 40 participants with MCI due to AD to ensure ≥ 30 participants, assuming a 25% missing data rate. To balance the number of participants among the three diagnostic groups, we recruited 120 participants (40 with MCI due to AD, 40 with mild ADD, and 40 HCs).

### 2.4 Data Collection Procedures

For participants with MCI due to AD or mild ADD, the most recent clinical diagnosis and age were confirmed from clinical records before the study was explained. After written informed consent was obtained, baseline assessments were conducted within 3 months (see Measures subsection). Participants who did not meet the inclusion criteria for these assessments (e.g., CDR global score other than 0.5, 1) were excluded from the analysis.

For HCs, written informed consent was obtained after the study was explained, and baseline assessments were then performed to determine eligibility using the predefined criteria described to the participants. HCs who did not meet these criteria were excluded from the analysis.

To test inter‐rater and test–retest reliabilities, the ADCS‐MCI‐ADL‐J was administered to participants with MCI due to AD at three time points: baseline, 1–2 weeks later, and 1–2 months later. Three occupational therapists and two neuropsychologists performed ADL assessments. The same interviewer conducted the baseline and third interviews. A different interviewer conducted the second interview. The first interviewer administered the other ADL scales of the PSMS, Lawton IADL, and DAD at baseline to verify the validity of the ADCS‐MCI‐ADL‐J. For participants with ADD and HC, all four ADL assessment scales were administered only once at baseline by the same interviewer.

For the ADL assessment, participants with MCI due to AD and mild ADD were assessed through interviews with their informants or caregivers, whereas HC participants responded to the interview. Interviews were conducted either in person or via telephone, as convenient for the interviewers or participants. For neuropsychiatric symptoms, the Neuropsychiatric Inventory Questionnaire (NPI‐Q) was administered only to participants with MCI due to AD or mild ADD, not to HCs.

### 2.5 ADCS‐MCI‐ADL

#### 2.5.1 Scale Characteristics

The ADCS‐MCI‐ADL scale is based on semi‐structured interviews with informants/caregivers [3]. This semi‐structured interview enables interviewers to consistently collect essential information, although the assessment requires more time (approximately 20–30 min) than simple questionnaires. The ADCS‐MCI‐ADL can be administered by clinical professionals involved in dementia care, such as occupational therapists or neuropsychologists. Interviewers can refer to the instructions or guidelines during the assessment.

This scale evaluates independence in ADL and accuracy or errors for some tasks during the past 4 weeks. It consists of 18 scored items and six additional items that are not included in the total score and are considered potentially difficult for patients with MCI. The items mainly assess advanced ADL (AADL), such as leisure activities or social interaction, and IADL, such as checking bank balance, keeping appointments, or preparing meals or snacks, and some BADL, such as dressing. The total score ranges from 0 to 53. Higher scores indicate better ADL.

#### 2.5.2 Translation Procedure

We translated the ADCS‐MCI‐ADL into Japanese following the International Society for Pharmacoeconomics and Outcomes Research (ISPOR) task force guidelines [19]. Permission for translation was obtained from the original author of the ADCS‐MCI‐ADL beforehand. Forward translation was conducted independently by two occupational therapists with PhDs specializing in ADL assessment. Two independent forward translations were considered for cultural differences and combined into a single Japanese version by a multidisciplinary research team. This version was then back‐translated into English by a bilingual expert, then the original author reviewed its conceptual equivalence with the original scale. During the cognitive debriefing, the provisional Japanese version was tested on a small sample to check understandability and interpretation. Finally, the translated scale was proofread.

### 2.6 Measures

Data on demographic characteristics, dementia severity, cognitive function, neuropsychiatric symptoms, and ADL were collected. All assessments were conducted within 3 months.

#### 2.6.1 Demographic Characteristics

Information on age, sex, living situation (alone or not), years of education, and disease duration was obtained from the medical records or participant interviews.

#### 2.6.2 Dementia Severity

Dementia severity was assessed using the CDR [20, 21]. Dementia stage was classified using CDR global scores as follows: 0, normal; 0.5, MCI or questionable dementia; 1, mild dementia; 2, moderate dementia; and 3, severe dementia. CDR global and sum of box (SoB) scores were obtained.

#### 2.6.3 Cognitive Function

Cognitive function was assessed using the MMSE‐J [15, 22]. The total score ranged from 0 to 30. Lower scores indicate greater cognitive impairment.

#### 2.6.4 Neuropsychiatric Symptoms

Neuropsychiatric symptoms were assessed using the NPI‐Q, a brief informant‐based questionnaire [23, 24]. The total score ranges from 0 to 30. Higher scores indicate more severe neuropsychiatric symptoms.

#### 2.6.5 ADL

ADL assessments were conducted using the ADCS‐MCI‐ADL‐J, PSMS, Lawton IADL, and DAD scales, which are generally used for patients with MCI or dementia in Japan.

The PSMS and Lawton IADL were developed as brief functional assessments for older adults [25, 26]. The PSMS is a six‐item scale that assesses BADL, such as toileting and feeding, with a total score ranging from 0 to 6. The Lawton IADL is an eight‐item scale that assesses IADL, such as shopping and food preparation, with a total score ranging from 0 to 8. For the Lawton IADL scale, the same assessment procedure was applied to all participants regardless of sex. Higher scores indicated better ADL on both scales. The DAD was developed as a functional scale for individuals with AD [27, 28]. It assesses both IADL and BADL and consists of 40 items. The total score ranged from 0 to 40 and was converted into a percentage score. For these assessment scales, higher scores indicate better ADL function.

Scoring of activities that were not routinely performed differed across the ADL measures. The Lawton IADL and PSMS assess capability, distinguishing inability to perform the ADL (0 points) from simply not performing the ADL despite retaining ability (1 point), based on the caregiver's perspective when assessing patients. In contrast, the ADCS‐MCI‐ADL‐J and DAD assess daily performance status and assign the same score for routine non‐performance regardless of the underlying reason, such as cognitive decline, premorbid habits, or personal lifestyle. However, in the DAD, items that subjects have never performed in their lives are rated as not applicable (NA) and excluded from the total score calculation.

### 2.7 Statistical Analysis

Descriptive statistics were calculated for all variables according to the three diagnostic groups. Group differences were examined using the Kruskal–Wallis or chi‐square test. When significant group differences were found, post hoc pairwise comparisons were performed using the Mann–Whitney *U* test or chi‐square test with Bonferroni correction.

#### 2.7.1 Reliability

To evaluate the reliability of the ADCS‐MCI‐ADL‐J, its internal consistency, inter‐rater reliability, and test–retest reliability were analysed. Internal consistency was assessed in all participants, while inter‐rater and test–retest reliabilities were assessed only in the MCI due to AD group.

Internal consistency was examined using Cronbach's *α*, with *α* ≥ 0.70 considered acceptable [29]. Intraclass correlation coefficient (ICC) was used to examine inter‐rater reliability [ICC (2, 1)] and test–retest reliability [ICC (1, 2)] for the total score (< 0.50 poor; 0.50–0.75, moderate; 0.75–0.90, good; > 0.90, excellent) [29]. ICC ≧ 0.70 was predefined as indicating acceptable reliability [30]. Agreement for individual sub‐item was examined using the Cohen's kappa or weighted kappa statistics with quadratic weighting and interpreted using Landis and Koch's rule (0.00–0.20, slight; 0.21–0.40, fair; 0.41–0.60, moderate; 0.61–0.80, substantial; 0.81–1.00, almost perfect) [31], while also checking the rate of participants who achieved the full score for each sub‐item. As a supplementary analysis, Gwet's Agreement Coefficients (AC) 1 or 2 with quadratic weighting was also calculated for all sub‐items to calculate the degree of score agreement for binary and ordinal variables, respectively [32], and interpreted using Landis and Koch's rule (0.00–0.20, slight; 0.21–0.40, fair; 0.41–0.60, moderate; 0.61–0.80, substantial; 0.81–1.00, almost perfect) [31]. This method is less influenced by prevalence and skew data than kappa statistics.

#### 2.7.2 Validity

Discriminative validity was analysed using a receiver operating characteristic (ROC) curve to distinguish the MCI due to AD groups from the HC and mild AD groups. The cutoff point was calculated with sensitivity and specificity using the Youden index. As a supplementary analysis, differences in sub‐item scores were also examined among the three diagnostic groups using the Kruskal–Wallis and post hoc Mann–Whitney *U* tests with Bonferroni correction.

Concurrent validity was examined using correlations between the ADCS‐MCI‐ADL‐J and other ADL assessment scales. Correlations among the ADL scales were analysed using Spearman's rank correlation coefficients.

Convergent validity was examined based on correlations between ADCS‐MCI‐ADL‐J scores and other clinical variables. The correlations among ADCS‐MCI‐ADL‐J, CDR‐SoB, and MMSE‐J were analysed in the total sample, while the correlation between ADCS‐MCI‐ADL‐J and NPI‐Q was analysed in patients with MCI due to AD and mild ADD.

Structural validity was assessed by exploratory factor analysis (EFA) using the baseline 23 scored items data of all participants. The suitability of the data for factor analysis was examined using the Kaiser–Meyer–Olkin measure of sampling adequacy and Bartlett's test of sphericity. The number of factors was determined using a parallel analysis. The EFA was performed using principal axis factoring with Promax rotation. Items with factor loadings ≥ 0.40 were considered to load on a factor.

#### 2.7.3 Interpretability

The distribution of total scores in each group for all ADL scales was checked for ceiling effects. Ceiling effects were considered present if > 15% of the respondents achieved the maximum score [29].

Statistical analyses were performed using SPSS version 29.0 (IBM Corp., USA) and R software version 4.6.1 (R Foundation for Statistical Computing, Austria). The Gwet's AC and parallel analysis were calculated using R; all other tests were performed using SPSS. Statistical significance was set at *p* < 0.05.

## 3 Results

### 3.1 Characteristics of Participants and Comparison of the Three Diagnosis Groups

Figure 1 shows a flowchart of the study participant selection. A total of 120 participants were enrolled in this study (40 each HCs, MCI due to AD, and mild ADD groups). Of them, 18 were excluded for the following reasons: missing baseline assessment data (*n* = 5), unstable health status (*n* = 3), not fulfilling the study criteria (*n* = 9), or withdrawal of consent (*n* = 1). Table 1 summarizes demographic and clinical characteristics of the remaining 102 participants (30 HCs, 37 with MCI due to AD, and 35 with mild ADD). Approximately 60% of participants were female (58.8%). The overall mean age (SD) was 72.6 (9.1) years. Most participants (91.2%) lived with their family. The mean years of education (SD) was 13.4 (2.5) years. Significant differences were observed between the diagnostic groups in terms of disease duration, CDR, MMSE‐J, and the four ADL scales (ADCS‐MCI‐ADL‐J, PSMS, Lawton IADL, and DAD). Demographic characteristics did not differ among the three groups. NPI‐Q scores did not differ significantly between the groups with MCI due to AD and mild ADD.

**FIGURE 1:**
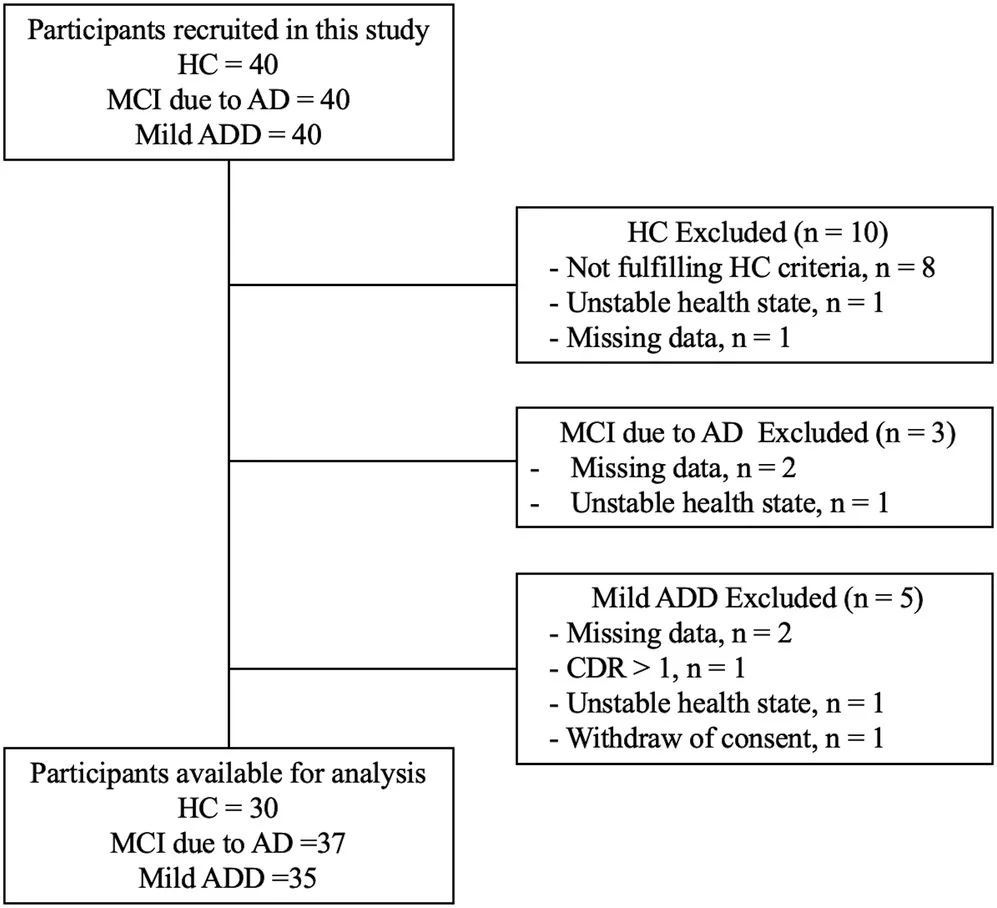
Flow chart of the study participants who met the inclusion/exclusion criteria.

**TABLE 1:** Characteristics of the study participants.

Variable	HC group (*n* = 30)	MCI due to AD group (*n* = 37)	Mild AD group (*n* = 35)	*p*
Age (years)	69.5 (8.8)	73.2 (9.5)	74.6 (8.5)	0.079^a^
Sex (male:female)	13:17	14:23	15:20	0.874^b^
Living alone (yes)	0	6	3	0.067^b^
Education history	13.8 (2.1)	13.5 (2.5)	13.1 (2.8)	0.584^a^
Disease duration	—	3.0 (1.9)	4.9 (3.4)	0.007^c^
CDR global (0:0.5:1)	30:0:0	0:37:0	0:6:29	< 0.001^b^
CDR SoB	0.0 (0.0)	2.6 (1.0)	5.7 (1.4)	< 0.001^a^, A > B > C
MMSE‐J score	29.5 (0.7)	24.7 (3.0)	19.3 (3.7)	< 0.001^a^, A > B > C
NPI‐Q score	—	2.3 (2.5)	3.5 (3.8)	0.145^c^
ADCS‐MCI‐ADL‐J score	48.9 (3.8)	41.3 (4.6)	32.4 (7.2)	< 0.001^a^, A > B > C
PSMS score	6.0 (0.2)	5.8 (0.5)	5.3 (0.8)	< 0.001^a^, A, B > C
Lawton IADL score	7.8 (0.5)	6.5 (1.3)	4.8 (1.6)	< 0.001^a^, A > B > C
DAD score (%)	97.7 (2.2)	89.2 (9.7)	70.5 (14.4)	< 0.001^a^, A > B > C

*Note:* A, HC; B, MCI due to AD; C, Mild ADD.

Abbreviations: AD, Alzheimer's disease; ADCS‐MCI‐ADL‐J, Japanese version of the Alzheimer's Disease Cooperative Study—Activities of Daily Living in Mild Cognitive Impairment; CDR, Clinical Dementia Rating; CDR SoB, Clinical Dementia Rating Sum of Boxes; DAD, Disability Assessment for Dementia; HC, healthy control; IADL, Instrumental Activities of Daily Living; MCI, mild cognitive impairment; MMSE‐J, Japanese version of the Mini‐Mental State Examination; NPI‐Q, Neuropsychiatric Inventory Questionnaire; PSMS, Physical Self‐Maintenance Scale.

^a^ Kruskal–Wallis test; post hoc test, Mann–Whitney *U* test with Bonferroni correction.

^b^ Chi‐square test.

^c^ Mann–Whitney *U* test.

### 3.2 Reliability of the ADCS‐MCI‐ADL‐J

The Cronbach's α for the ADCS‐MCI‐ADL‐J was 0.893, indicating acceptable internal consistency.

Table 2 summarizes the inter‐rater and test–retest reliability results for the sub‐items. Regarding inter‐rater reliability, 33 participants with MCI due to AD were included in the analysis. The ICC (2, 1) was 0.917 (*p* < 0.001), indicating excellent inter‐rater reliability. Twenty sub‐items showed significant agreement with moderate to almost perfect levels (sub‐items 4, 5, 6, 7, 8, 10, 11, 12a, 12d, 13a, 13b, 13c, 14c, 15b, 16a, 17, 19, 20, 21, and 23). One sub‐item showed significant fair agreement (sub‐item 14b). The other 12 sub‐items showed no significant agreement (items 1, 2, 3, 9, 12b, 12c, 14a, 15a, 16b, 16c, 18, and 22). In contrast, Gwet's AC 1/AC2 showed significant agreement and moderate‐to‐almost perfect levels for all sub‐items.

**TABLE 2:** Inter‐rater and test–retest reliabilities of ADCS‐MCI‐ADL‐J items.

Item	Inter‐rater reliability (*n* = 33)			Test–retest reliability (*n* = 31)		
	κ Wκ	Full score rate 1st/2nd	AC1 AC2	κ Wκ	Full score rate 1st/3rd	AC1 AC2
1. Finding belongings at home^b^	0.249	72.7%/69.7%	0.789***	0.708***	71.0%/77.4%	0.946***
2. Choosing the clothes for the day^b^	0.000	93.9%/100%	0.993***	0.652***	96.8%/93.5%	0.996***
3. Changing clothes^b^	N/A	100%/100%	1.00***	N/A	100%/100%	1.00***
4. Cleaning the room^b^	0.506**	72.7%/72.7%	0.711***	0.744***	77.4%/77.4%	0.874***
5. Checking the bankbook balance^b^	0.455**	51.5%/63.6%	0.472**	0.713***	58.1%/54.8%	0.720***
6. Writing^b^	0.545**	27.3%/24.2%	0.822***	0.114	29.0%/22.6%	0.731***
7. Doing the laundry^b^	0.897***	69.7%/72.7%	0.933***	0.862***	71.0%/77.4%	0.920***
8. Keeping promises^b^	0.605***	0.0%/3.0%	0.963***	0.290	0.0%/3.2%	0.933***
9. Using the phone^b^	0.005	6.1%/12.1%	0.905***	0.125	6.5%/3.2%	0.930***
10. Preparing meals and snacks^b^	0.879***	93.9%/87.9%	0.992***	1.00***	93.5%/93.5%	1.00***
11. Going out/travelling far^b^	0.506***	93.9%/81.8%	0.945***	0.878***	93.5%/87.1%	0.991***
12. Talking about recent events						
a. Local, national or international events^a^	0.455**	84.8%/81.8%	0.790***	0.426*	87.1%/87.1%	0.834***
b. Incident outside the home^a^	−0.012	78.8%/84.8%	0.569***	0.456**	77.4%/87.1%	0.772***
c. Domestic events^a^	0.330	78.8%/81.8%	0.690***	0.285	83.9%/83.9%	0.735***
d. A conversation without repeating^a^	0.654***	27.3%/36.4%	0.732***	0.530**	29.0%/29.0%	0.671***
13. Reading books						
a. Choose something to read^a^	0.742***	78.8%/75.8%	0.860***	0.763***	80.6%/87.1%	0.912***
b. Talk about the content while reading^a^	0.694***	27.3%/27.3%	0.799***	0.575***	22.6%/41.9%	0.656***
c. Talk about the content after reading^a^	0.670***	24.2%/24.2%	0.808***	0.663**	25.8%/25.8%	0.791***
14. Watching television						
a. Change the channel^a^	−0.031	97.0%/97.0%	0.939***	0.783*	93.5%/90.3%	0.962***
b. Talk about TV programmes while watching^a^	0.343*	72.7%/78.8%	0.617***	0.561**	77.4%/74.4%	0.745***
c. Talk about the TV programme within a day^a^	0.426*	42.4%/33.3%	0.485**	0.483**	48.4%/48.4%	0.484**
15. Going shopping						
a. Choose the appropriate item^a^	0.225	78.8%/75.8%	0.580***	0.815***	77.4%/77.4%	0.901***
b. Pay for the items^a^	0.784***	93.9%/90.9%	0.965***	0.635***	93.5%/87.1%	0.922***
16. Staying safe alone						
a. Spend alone away from home^a^	0.531***	93.9%/84.8%	0.888***	0.783***	93.5%/90.3%	0.962***
b. Spend more than an hour alone at home^a^	N/A	100%/100%	1.00***	N/A	100%/100%	1.00***
c. Spend alone less than an hour at home^a^	N/A	100%/100%	1.00***	N/A	100%/100%	1.00***
17. Using electric products^b^	0.712***	87.9%/81.8%	0.990***	0.557***	87.1%/90.3%	0.970***
18. Engaging in recreation or hobby^b^	0.280	75.8%/75.8%	0.677***	0.675***	77.4%/80.6%	0.891***
19. Driving an automobile^b^	0.927***	12.1%/6.1%	0.989***	0.874***	9.7%/6.5%	0.947***
20. Regularly taking medications^b^	0.888***	51.5%/48.9%	0.936***	0.575***	48.4%/51.6%	0.739***
21. Completing complex daily activities^b^	0.514**	54.5%/60.6%	0.593***	0.501**	51.6%/51.6%	0.593***
22. Spontaneously managing complex daily activities^b^	0.322	51.5%/51.5%	0.686***	0.520**	45.2%/54.8%	0.811***
23. Time spent on complex routine activities^b^	0.499**	27.3%//33.3%	0.693***	0.444**	29.0%/22.6%	0.694***

Abbreviations: κ, Kappa; Wκ, Weighted kappa; AC1, Gwet's Agreement Coefficients 1; AC2, Gwet's Agreement Coefficients 2.

^a^ Kappa statistics, Gwet's AC1, *p < 0.05, **p < 0.01, ***p < 0.001.

^b^ Weighted kappa statistics, Gwet's AC2, *p < 0.05, **p < 0.01, ***p < 0.001.

Thirty‐one participants were included in the analysis for test–retest reliability. Similarly, the ICC (1, 2) was 0.915 (*p* < 0.001), indicating excellent test–retest reliability. Many sub‐items showed moderate to almost perfect agreement (items 1, 2, 4, 5, 7, 10, 11, 12a, 12b, 12d, 13a, 13b, 13c, 14a, 14b, 14c, 15a, 15b, 16a, 17, 18, 19, 20, 21, 22, and 23). The other seven sub‐items showed no significant agreement (sub‐items 3, 6, 8, 9, 12c, 16b, 16c). In contrast, Gwet's AC 1/AC2 showed significant agreement and moderate‐to‐almost perfect levels for all sub‐items.

### 3.3 Validity of the ADCS‐MCI‐ADL‐J

Figure 2 shows the ROC curves of the ADCS‐MCI‐ADL‐J, which distinguished MCI due to AD from HC and mild ADD. The areas under the curve (AUCs) were 0.937 (*p* < 0.001; 95% confidence interval, 0.869–1.00) and 0.859 (*p* < 0.001, 95% confidence interval, 0.775–0.942) for the MCI due to AD group versus HCs and mild ADD group, respectively. The optimal cutoff scores to distinguish MCI due to AD from HC and mild ADD were 46/47 points (sensitivity, 89.2%; specificity, 90.0%) and 36/37 points (sensitivity, 89.2%; specificity, 68.6%), respectively. Table 3 presents a comparison of the ADCS‐MCI‐ADL‐J sub scores among the three groups. Significant differences between MCI due to AD and HC were observed mainly in the AADL items (items 6, 12, 13, 14, 18, 19, 22, and 23) and some IADL items (items 5, 8, and 9). Significant differences between MCI due to AD and mild ADD were also observed mainly in IADL items (items 4, 5, 8, 10, 11, 15, 17, and 20) and some BADL items (items 2 and 16).

**FIGURE 2:**
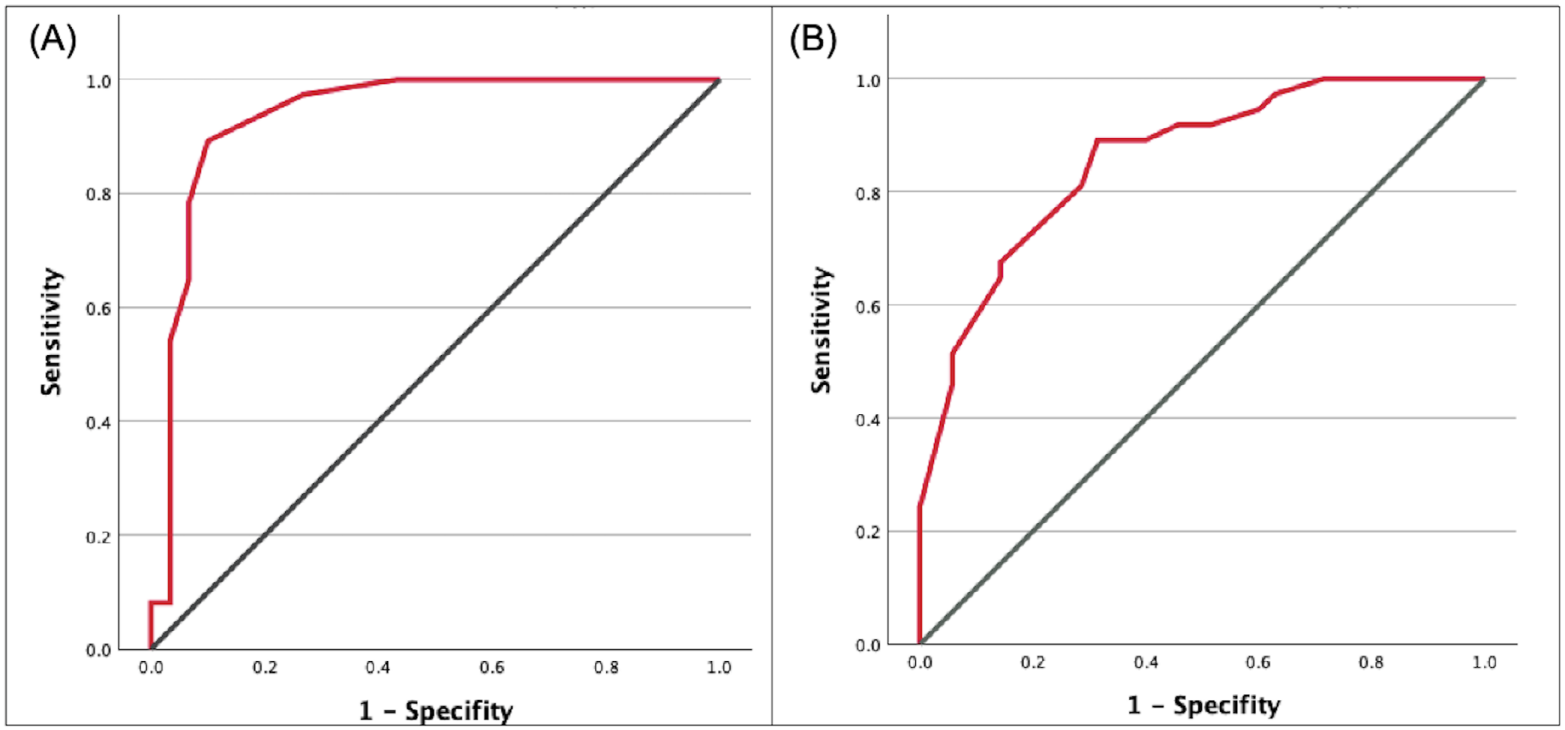
ROC curve differentiating between MCI due to AD from HC and mild ADD. (A) results for MCI due to AD versus HC. The AUC was 0.937 (*p* < 0.001; 95% confidence interval, 0.869–1.00), with a cutoff point of 46/47. (B) results for MCI due to AD versus mild ADD. The AUC was 0.859 (*p* < 0.001, 95% confidence interval, 0.775–0.942) with a cutoff point of 36/37.

**TABLE 3:** Comparison of ADCS‐MCI‐ADL sub‐item scores among the three groups.

Sub‐item	Score range	HC group (*n* = 30)	MCI due to AD group (*n* = 37)	Mild AD group (*n* = 35)	*p*
1. Finding belongings at home	0–3	3.0 (0.0)	2.6 (0.8)	2.3 (0.8)	< 0.001, A > C
2. Choosing the clothes for the day	0–3	3.0 (0.0)	2.9 (0.3)	2.5 (0.7)	< 0.001, A, B > C
3. Changing clothes	0–4	4.0 (0.0)	4.0 (0.0)	4.0 (0.2)	0.384
4. Cleaning the room	0–2	1.7 (0.7)	1.5 (0.8)	1.0 (1.0)	< 0.001, A, B > C
5. Checking the bankbook balance	0–2	1.9 (0.4)	1.1 (1.0)	0.4 (0.8)	< 0.001, A > B > C
6. Writing	0–2	1.7 (0.5)	1.2 (0.5)	1.0 (0.6)	< 0.001, A > B, C
7. Doing the laundry	0–2	1.5 (0.9)	1.5 (0.9)	1.1 (0.9)	0.126
8. Keeping promises	0–3	2.4 (0.5)	1.8 (0.5)	1.1 (0.7)	< 0.001, A > B > C
9. Using the phone	0–4	3.4 (0.9)	2.1 (0.6)	1.7 (1.1)	< 0.001, A > B, C
10. Preparing meals and snacks	0–3	2.8 (0.8)	2.8 (0.7)	2.1 (1.3)	< 0.001, A, B > C
11. Going out/travelling far	0–3	3.0 (0.0)	2.9 (0.5)	2.1 (0.9)	< 0.001, A, B > C
12. Talking about recent events	0–4	3.8 (0.4)	2.7 (0.9)	2.0 (1.1)	< 0.001, A > B, C
13. Reading books	0–3	2.0 (1.0)	1.3 (1.0)	0.8 (0.9)	< 0.001, A > B, C
14. Watching television	0–3	2.7 (0.7)	2.1 (0.8)	1.8 (0.8)	< 0.001, A > B, C
15. Going shopping	0–2	2.0 (0.2)	1.7 (0.6)	0.9 (0.8)	< 0.001, A, B > C
16. Staying safe alone	0–3	3.0 (0.0)	3.0 (0.2)	2.4 (0.7)	< 0.001, A, B > C
17. Using electric products	0–4	3.9 (0.7)	3.8 (0.7)	3.2 (1.3)	< 0.001, A, B > C
18. Engaging in recreation or hobby	0–3	3.0 (0.0)	2.3 (1.2)	2.0 (1.2)	< 0.001, A > B, C
19. Driving an automobile	0–3	1.7 (1.3)	0.7 (1.1)	0.1 (0.3)	< 0.001, A > B, C
20. Regularly taking medications	0–4	2.9 (1.8)	2.9 (1.4)	1.8 (1.1)	< 0.001, A, B > C
21. Completing complex daily activities	0–3	2.8 (0.8)	2.2 (1.1)	1.3 (1.2)	< 0.001, A, B > C
22. Spontaneously managing complex daily activities	0–3	2.9 (0.4)	2.2 (0.9)	1.6 (0.8)	< 0.001, A > B > C
23. Time spent on complex routine activities	0–3	2.7 (0.5)	1.9 (0.9)	1.5 (0.7)	< 0.001, A > B, C

*Note:* Kruskal–Wallis test, post hoc test; Mann–Whitney U test with Bonferroni correction. A, HC; B, MCI due to AD; C, Mild ADD.

Table 4 shows the correlations between the ADCS‐MCI‐ADL‐J and other clinical variables. ADCS‐MCI‐ADL‐J scores were significantly correlated with other ADL scale scores (PSMS: ρ = 0.524, *p* < 0.001; Lawton IADL: ρ = 0.827, *p* < 0.001; DAD: ρ = 0.886, *p* < 0.001). ADCS‐MCI‐ADL‐J scores were significantly correlated with the CDR‐SoB, MMSE‐J, and NPI‐Q scores (ρ = −0.859, *p* < 0.001; ρ = 0.780, *p* < 0.001; ρ = −0.412, *p* < 0.001; respectively); specifically, higher ADCS‐MCI‐ADL‐J scores were associated with less severe dementia, better cognition, and milder neuropsychiatric symptoms.

**TABLE 4:** Correlations between ADCS‐MCI‐ADL‐J score and other assessment scale scores.

	ADCS‐MCI‐ADL‐J	PSMS	Lawton IADL	DAD	CDR SoB	MMSE‐J	NPI‐Q
ADCS‐MCI‐ADL‐J		0.524***	0.827***	0.886***	−0.859***	0.780***	−0.412***
PSMS			0.518***	0.539***	−0.490***	0.427***	−0.262*
Lawton IADL				0.838***	−0.772***	0.670***	−0.166
DAD					−0.854***	0.781***	−0.373**
CDR SoB						−0.884***	0.273*
MMSE‐J							−0.202
NPI‐Q							

*Note:* Spearman's rank correlation. *p < 0.05, **p < 0.01, ***p < 0.001.

Abbreviations: ADCS‐MCI‐ADL‐J, Japanese version of the Alzheimer's Disease Cooperative Study—Activities of Daily Living in Mild Cognitive Impairment; CDR, Clinical Dementia Rating; CDR SoB, Clinical Dementia Rating Sum of Boxes; DAD, Disability Assessment for Dementia; IADL, instrumental activities of daily living; MMSE‐J, Japanese version of the Mini‐Mental State Examination; NPI‐Q, Neuropsychiatric Inventory Questionnaire; PSMS, Physical Self‐Maintenance Scale.

Table 5 presents the results of the EFA. The Kaiser–Meyer–Olkin measure was 0.848, and Bartlett's test was significant (χ^2^ = 995.352, df = 253, *p* < 0.001), indicating that the correlation matrix was suitable for factor analysis. Although parallel analysis indicated a four‐factor solution, a three‐factor solution was ultimately retained because the fourth factor contained too few items (two items) to be considered meaningful (loading ≥ |0.40|). Moreover, two items (item 11, going out/travelling far and item 16, staying safe alone) did not form an interpretable construct, making it difficult to assign a meaningful factor name (Supplementary Table 1). These three factors account for 33.4%, 9.1%, and 6.7% of the variance, respectively. Factor 1 comprised seven items (sub‐items 5, 15, 18, 19, 21, 22, and 23), with factor loadings from 0.470 to 0.870. Factor 2 comprised six items (sub‐items 1, 9, 12, 13, 14, and 16), with factor loadings from 0.449 to 0.757. Factor 3 comprised five items (sub‐items 2, 4, 7, 10, and 17) with factor loadings ranging from 0.434 to 0.746. These factors were summarized as follows: ‘Complex activity’, ‘Leisure/Social activity’, and ‘Housework activity’, respectively.

**TABLE 5:** Factor structure of the ADCS‐MCI‐ADL‐J.

	Factor 1 ‘Complex activity’	Factor 2 ‘Leisure/social activity’	Factor 3 ‘Housework activity’
21. Completing complex daily activities	0.**870**	−0.203	0.159
22. Spontaneously managing complex daily activities	0.**783**	−0.141	0.071
23. Time spent on complex routine activities	0.**673**	0.020	0.006
15. Going shopping	0.**595**	0.059	0.209
18. Engaging in recreation or hobby	0.**555**	−0.093	−0.153
5. Checking the bankbook balance	0.**509**	0.225	0.032
19. Driving an automobile	0.**470**	0.330	−0.330
20. Regularly taking medications	0.364	0.173	−0.097
14. Watching television	−0.098	0.**757**	−0.059
16. Staying safe alone	−0.124	0.**619**	0.101
12. Talking about recent events	0.223	0.**569**	−0.026
9. Using the phone	0.122	0.**567**	0.032
1. Finding belongings at home	−0.011	0.**475**	0.058
13. Reading books	0.201	0.**449**	−0.126
3. Changing clothes	−0.149	0.397	0.121
8. Keeping promises	0.391	0.396	0.028
11. Going out/travelling far	0.282	0.379	0.013
6. Writing	0.181	0.342	0.104
7. Doing the laundry	−0.056	−0.203	0.**746**
10. Preparing meals and snacks	−0.220	0.282	0.**708**
17. Using electric products	0.216	−0.039	0.**672**
4. Cleaning the room	0.027	0.141	0.**516**
2. Choosing the clothes for the day	−0.031	0.343	0.**434**
**Proportion of variance explained**	33.4%	9.1%	6.7%
**Cumulative proportion of variance explained**	33.4%	42.5%	49.2%

*Note:* Bold and boxed values indicate items belonging to the same factor.

### 3.4 Interpretability of the ADCS‐MCI‐ADL‐J

#### 3.4.1 ADL Score Distributions

Figure 3 shows the distribution of the ADL scale scores for each group. There was no ceiling effect among HCs in the ADCS‐MCI‐ADL‐J. For patients with MCI due to AD, no ceiling effects were observed in the DAD or ADCS‐MCI‐ADL‐J groups, whereas ceiling effects were observed in the PSMS and Lawton IADL groups. The percentages of participants with the maximum possible scores were 0.0%, 81.1%, 29.7%, and 8.1% for the ADCS‐MCI‐ADL‐J, PSMS, Lawton IADL, and DAD, respectively. For participants with mild ADD, the ceiling effect did not occur in any of the three ADL scales except the PSMS.

**FIGURE 3:**
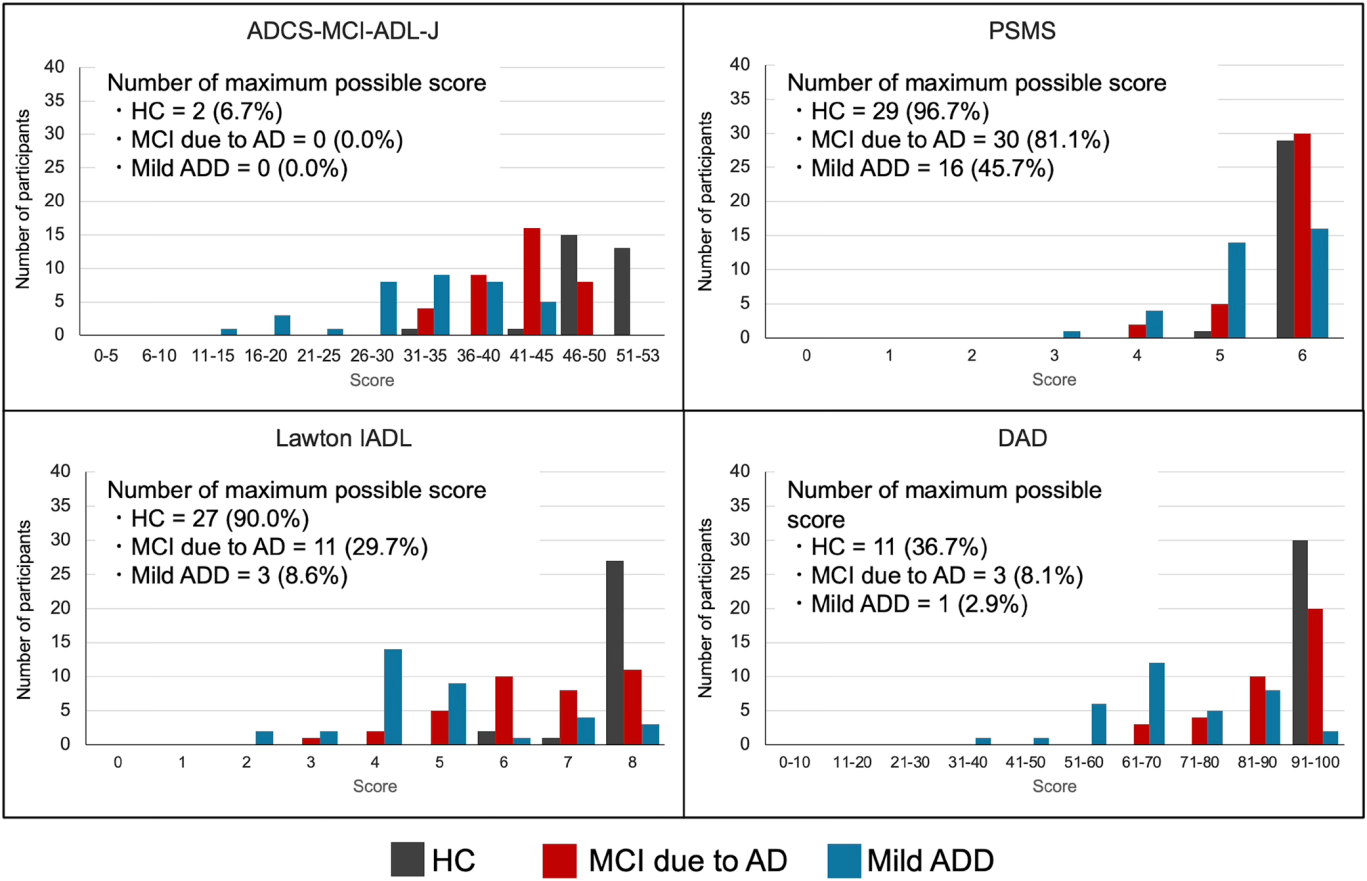
The score distribution of four ADL assessment scales. ADCS‐MCI‐ADL‐J, Japanese version of the Alzheimer's Disease Cooperative Study Scale for Activities of Daily Living in Mild Cognitive Impairment; PSMS, Physical Self‐Maintenance Scale; Lawton IADL, Instrumental Activities of Daily Living; DAD, Disability Assessment for Dementia.

## 4 Discussion

This study examined the psychometric characteristics of the ADCS‐MCI‐ADL‐J, an ADL assessment scale adapted for patients with MCI. This scale exhibited good reliability, validity, and interpretability in a sample population of patients with MCI due to AD. Compared to other existing Japanese ADL scales, the ADCS‐MCI‐ADL‐J appears more useful for ADL assessment in the MCI stage.

Our results also showed that the ADCS‐MCI‐ADL‐J has good internal consistency, inter‐rater reliability, and test–retest reliability. In a previous study on the validation of the ADCS‐MCI‐ADL, the Cronbach's α was 0.85, indicating acceptable internal consistency [33]. Consistent with this finding, the ADCS‐MCI‐ADL‐J also showed acceptable internal consistency, although the present study included not only MCI due to AD but also HC and mild ADD, unlike the previous study. Concerning inter‐rater reliability and test–retest reliability, both ICC coefficients were excellent in terms of the total score. However, many sub‐items did not reach slight to fair agreement among raters or between the two assessment time points. This result could be explained by the statistical limitation of the kappa/weighted‐kappa coefficient when response distributions are highly skewed, referring to the kappa paradox [32]. Indeed, high full score rates were observed for most sub‐items at multiple assessment points. Importantly, Gwet's AC1 and 2 showed significant agreement at moderate to almost perfect levels for all subitems. Based on these results, inter‐rater and test–retest reliability in the sub‐items would also be positive, although the statistical test for these reliabilities has been suggested to use kappa or weighted kappa statistics according to the COSMIN checklist [18].

Concerning the validity of the ADCS‐MCI‐ADL‐J, the ROC curves significantly distinguished MCI due to AD from HC and mild ADD, indicating good discriminative validity. Notably, the AUC was larger for the distinction between MCI due to AD and HC compared to MCI due to AD versus mild ADD. This suggests that the ADCS‐MCI‐ADL‐J is useful for ADL assessment at an earlier stage, such as in the healthy and MCI stages. Our findings support several previous reports of good capability of ADCS‐MCI‐ADL to discriminate between MCI and HCs [6, 34]. Supplemental analysis of sub‐items revealed significant differences mainly in AADL and some IADL for comparison between MCI due to AD and HC. Given the general course in which ADL begins to decline first at a more complex level [35], the observed differences in IADL performance between the HC and MCI groups are reasonable and contribute to the discriminative capability of ADCS‐MCI‐ADL. However, given the methodological difference of ADL assessment between HC group (self‐report) and patient group (informant‐report), caution is required to interpret this finding. This discrepancy might introduce measurement bias, such as differences in recall of ADL performance or social desirability bias, potentially leading to an overestimation of the discriminative ability between the HC and MCI groups.

The ADCS‐MCI‐ADL‐J was significantly correlated with other existing ADL scales, supporting its concurrent validity. This finding suggests that the ADCS‐MCI‐ADL‐J has a capability similar to the existing scales in detecting ADL decline in individuals across MCI spectra. As hypothesized, the ADCS‐MCI‐ADL‐J score was also significantly correlated with various clinical variables, indicating good convergent validity of the ADCS‐MCI‐ADL‐J. The ADL score was significantly associated with dementia severity (CDR‐SoB score) or cognitive dysfunction (MMSE‐J score), as these relationships have been extensively reported previously [33, 36, 37]. In this study, the ADCS‐MCI‐ADL‐J score was also associated with neuropsychiatric symptoms (NPI‐Q score), although the correlation was weak to moderate. Some neuropsychiatric symptoms, including apathy or depression, are known to occur even in the early stages of MCI or mild dementia [38, 39]. Additionally, several studies have reported that cognitive dysfunction as well as neuropsychiatric symptoms could affect ADLs at the MCI stage [40, 41]. The ADCS‐MCI‐ADL‐J is likely to comprehensively capture ADL decline caused by several clinical problems, such as cognitive dysfunction or neuropsychiatric symptoms in the MCI stage.

EFA identified a three‐factor structure for the ADCS‐MCI‐ADL‐J, comprising complex, leisure/social, and housework activities. This finding may reflect the characteristics of the ADCS‐MCI‐ADL in assessing multiple levels of ADL, encompassing not only IADL, but also AADL [3]. However, the factor structure of the original ADCS‐MCI‐ADL has not yet been investigated [33], preventing comparison of this finding with other existing results. Further studies are needed to examine the factor structure of the original ADCS‐MCI‐ADL as well as other translated versions. Notably, some factors included items with relatively low factor loadings or items that appeared conceptually inconsistent with the corresponding factor. For example, finding belongings at home (Item 1) loaded on Leisure/Social Activity or Choosing clothes for the day (Item 2) loaded on Housework Activity. Therefore, caution is warranted when interpreting these findings, particularly regarding factor naming and item grouping. The present findings do not establish the clinical utility of the three factor‐based subscores at this stage. Further investigation is needed before the subscores can be used for domain‐specific clinical assessment.

Regarding interpretability, a ceiling effect did not occur in the ADCS‐MCI‐ADL‐J, whereas the score distributions of the other three ADL scales were higher. Interestingly, this scale can also assess the HC without a ceiling effect. The ADCS‐MCI‐ADL was originally adapted from the ADCS‐ADL for evaluating patients with MCI in more detail and consists of mainly IADL and some social activities [3]. This scale composition in the ADCS‐MCI‐ADL could increase the feasibility for thoroughly assessing the HCs, as well as patients with MCI, compared with other ADL scales.

This study had some limitations. First, the sample size of the MCI due to AD group was relatively small, although the reliability was acceptable. However, a larger sample may have contributed to improved reliability and validity of the ADCS‐MCI‐ADL‐J. Second, the psychometric properties verified in this study are reliability, validity, and interpretability. Future studies are required to investigate other properties that enhance the quality of the ADCS‐MCI‐ADL‐J. Finally, ADL assessment procedures differed between the patient and HC groups. ADL was assessed through interviews with informants in the patient group and through interviews with HCs. This difference in assessment procedures may have led to an overestimation of ADLs among HCs, although the eligibility of HCs was carefully evaluated. Therefore, the results, particularly the comparisons between the MCI due to AD group and HCs, should be interpreted with caution, although several previous studies have used different assessment procedures for patients and HCs [2, 35].

In conclusion, this study revealed that the ADCS‐MCI‐ADL‐J could be useful for ADL assessment in patients with MCI due to AD. Nonetheless, future studies should investigate other psychometric properties of this instrument. This scale may aid in detecting the subtle decline in ADL in patients with MCI due to AD and in measuring the outcome of ADL changes for clinical intervention.

## Funding

This study was supported by a Health, Labor, and Welfare Science Research Grant (grant number 22GB1001) and JSPS KAKENHI (grant number 21K17409).

## Ethics Statement

This study was approved by the University of Osaka Clinical Research Review Committee [23 274(T4)‐8].

## Consent

Written informed consent was obtained from patients and/or caregivers.

## Conflicts of Interest

The authors declare no conflicts of interest.

## Supporting information


**Supplementary Table 1** Four‐factor structure of the ADCS‐MCI‐ADL‐J.

## Data Availability

The data that support the findings of this study are available from the corresponding author (Daiki Ishimaru: d.ishimaru@psy.med.osaka-u.ac.jp) upon reasonable request.
