# Supplementary material for: Development of the Japanese Version of the Alzheimer's Disease Cooperative Study—Activities of Daily Living in Mild Cognitive Impairment Scale
Source: Psychogeriatrics. 2026 Oct 20;26(6):e70232. doi: 10.1111/psyg.70232 (PMC13683548; doi:10.1111/psyg.70232)
Supplement: Supplementary file 1 [file PSYG-26-0-s001.docx]

**Supplementary Table 1.** Four-factor structure of the ADCS-MCI-ADL-J

|  | **Factor 1** | **Factor 2** | **Factor 3** | **Factor 4** |
| --- | --- | --- | --- | --- |
| 21. Completing complex daily activities | **.864** | -.149 | .137 | -.047 |
| 22. Spontaneously managing complex daily activities | **.766** | -.085 | .061 | -.046 |
| 15. Going shopping | **.675** | -.093 | .151 | .210 |
| 23. Time spent on complex routine activities | **.640** | .088 | .017 | -.058 |
| 5. Checking the bankbook balance | **.521** | .158 | .011 | .133 |
| 18. Engaging in recreation or hobby | **.471** | .225 | -.070 | -.454 |
| 19. Driving an automobile | **.413** | .343 | -.301 | .041 |
| 8. Keeping promises | .376 | .370 | .040 | .086 |
| 20. Regularly taking medications | .362 | .121 | -.107 | .103 |
| 12. Talking about recent events | .084 | **.819** | .055 | -.176 |
| 14. Watching television | -.146 | **.730** | -.022 | .133 |
| 9. Using the phone | .092 | **.554** | .063 | .079 |
| 13. Reading books | .169 | **.429** | -.101 | .080 |
| 1. Finding belongings at home | -.021 | **.428** | .069 | .116 |
| 6. Writing | .135 | **.424** | .149 | -.064 |
| 3. Changing clothes | -.116 | .271 | .104 | .200 |
| 7. Doing the laundry | -.030 | -.099 | **.772** | -.186 |
| 10. Preparing meals and snacks | -.106 | .060 | **.649** | .305 |
| 17. Using electric products | .280 | -.053 | **.649** | -.005 |
| 4. Cleaning the room | .038 | .182 | **.530** | -.044 |
| 2. Choosing the clothes for the day | -.015 | .320 | **.440** | .064 |
| 11. Going out/traveling far | .427 | -.042 | -.158 | **.705** |
| 16. Staying safe alone | -.069 | .293 | .006 | **.601** |
| **Proportion of variance explained** | 33.4% | 9.1% | 6.7% | 6.1% |
| **Cumulative proportion of variance explained** | 33.4% | 42.5% | 49.2% | 55.3% |
